# Effect of Doping with Different Nb Contents on the Properties of CoCrFeNi High-Entropy Alloys

**DOI:** 10.3390/ma16196407

**Published:** 2023-09-26

**Authors:** Jingyu Zhang, Ke Xiong, Lin Huang, Bo Xie, Daping Ren, Chen Tang, Wei Feng

**Affiliations:** 1 School of Mechanical Engineering, Chengdu University, Chengdu 610106, China; zhangjingyu@stu.cdu.edu.cn (J.Z.); xiongke@stu.cdu.edu.cn (K.X.); 2 Sichuan Province Engineering Technology Research Center of Powder Metallurgy, Chengdu 610106, China; 3 Chengdu Tool Research Institute Co., Ltd., Chengdu 610100, China; xbmjq@163.com (B.X.); 18111283363@139.com (D.R.); ctritc415@163.com (C.T.)

**Keywords:** CoCrFeNi-Nbx HEAs, spark plasma sintering, high-energy ball milling, microstructure, mechanical properties

## Abstract

A series of five-element CoCrFeNi-Nbx (x = 0, 1, 3, 5, 7, and 9 wt%) high-entropy alloys were prepared using high-energy ball milling and discharge plasma sintering methods. Then, the effects of doping with Nb elements on the organization and properties of the CoCrFeNi HEAs were systematically investigated by tensile testing, hardness testing, and examining their micro-morphologies. The results show that with the addition of the Nb element, the lattice distortion of the alloy due to the large size of the Nb atoms causes the microstructure of CoCrFeNi HEAs to change from a single-phase FCC structure to a dual-phase structure of FCC and Laves. With the increase in the Nb content, the increase in the volume fraction of the hard and brittle Laves phase leads to the enhancement of the HEA’s tensile strength, yield strength, and hardness, and a decrease in plasticity. The Nb5 alloy showed the most excellent comprehensive performance, with a tensile strength, yield strength, and plasticity of 879.1 MPa, 491.8 MPa, and 39.8%, respectively, and all the properties were improved compared with those of the HEAs obtained by the arc melting method. The increase in the hardness of the HEAs was nearly proportional to the increase in the volume fraction of the Laves phase, which was the direct cause of the increase in the hardness of the HEA. Therefore, since the Laves phase is the direct cause of the increase in HEA hardness, the doping of CoCrFeNi HEAs with Nb can significantly improve the properties of HEAs.

## 1. Introduction

In recent years, the development of a new type of alloy, consisting of five or more equal or approximately equal metals, which possesses many excellent properties and characteristics, such as fracture resistance, tensile strength, corrosion resistance, and oxidation resistance, has attracted a great deal of attention from the scientific community. Since Cantor et al. [1] reported the design concept of high entropy alloys (HEAs) in 2004, scientists have carried out a lot of research on HEAs and found that HEAs can be composed of face-centered cubic (FCC), body-centered cubic (BCC), hexagonal close-packed (HCP), and topologically close-packed (TCP) phases [2,3]. Most of the past research used vacuum arc melting technology to prepare HEAs; the disadvantages of this method are the high cost and limited shape and size of samples, while the advantages of the discharge plasma sintering (SPS) process are very obvious: uniform heating, fast heating speed, low sintering temperature, short sintering time, high productivity, fine and uniform organization of the product, the ability to maintain the natural state of the raw material, the ability to obtain high-density materials, easy operation, etc. [4,5,6,7,8]. At the same time, it has been found that the SPS process possesses many properties: (1) Enhanced sintering: with this process, materials have been reported to sinter at lower temperatures, to higher densities, and with shorter dwell times, (2) improved or unique properties: materials sintered by the SPS process have been reported to possess better, or in some cases, unanticipated advantageous properties, and (3) new structures or phases: by using the SPS process, new microstructures or phases have been prepared [9]. Therefore, in this study, CoCrFeNi-Nbx (x = 0, 1, 3, 5, 7, and 9 wt%) HEAs were prepared by using high-energy ball milling and discharge plasma sintering methods, and CoCrFeNi HEAs were made to decrease their plasticity and increase their strength by the addition of the element Nb. Meanwhile, the experimental results showed that after the addition of the Nb element, a new Laves hard phase with an HCP structure was found in the HEA [10], and the proportion of the hard Laves phase gradually increased with the increase in Nb, which led to a large increase in the hardness and tensile strength of the alloy and a decrease in its plasticity [11]. Since the four elements Co, Cr, Fe, and Ni have very similar atomic sizes and their enthalpies of mixing are small, as shown in Table 1, they exhibit a face-centered cubic (FCC) single-phase solid solution structure with better plasticity and poorer strength after solid solution, while it was found that alloys with a face-centered cubic (FCC) structure tend to have better strength and poorer plasticity. Therefore, the composite microstructure can be changed to solve the problem of the contradictory strength and plasticity of the two, i.e., doping impurity atoms into the HEA makes the HEA produce a new BCC phase, which improves the strength by decreasing the plasticity in order to obtain alloys with more excellent properties [12,13,14,15,16,17]. Due to the very negative enthalpy of mixing between the metal element Nb and the elements Co, Cr, Fe, and Ni, it is easier to realize the solid solution strengthening and the formation of hard (Laves phase) and brittle phases [18,19,20,21]. For the CoCrFeNiNb9 HEA, its tensile strength and hardness can reach 893.2 MPa and 336 HV, respectively, while its elongation can be maintained above 11.8%. Observation of the tensile fracture reveals that the face-centered cubic matrix of the CoCrFeNi-Nbx HEAs is a ductile fracture and that the Laves phase is a brittle fracture [22], which provides strong support for the explanation of the changes in strength and toughness due to the increased niobium content.

## 2. Materials and Methods

In this experiment, equal mass ratios of CoCrFeNi powder (purity > 99.9 wt%) and Nb powder (purity > 99.9 wt%) were mixed in different mass ratios. The molds were sealed and flushed with argon gas (purity > 99.99%) at a flow rate of 2 L/min for ten minutes. The molds were placed in a high-energy ball mill (omnidirectional planetary ball mill PMQW4, Nanjing Chishun Science and Technology Development Co., Ltd., Nanjing, China) for mechanical alloying at 400 rpm for 30 min intervals, followed by a 10 min interruption to allow cooling. These steps were repeated until the mechanical alloying process reached 4 h. A total of 20 g of the prepared alloy powder was placed into a graphite mold with a diameter of 20 mm, and the sample was compacted using an indenter and carbon paper, pressurized at 30 MPa, and sintered by discharge plasma sintering equipment ((SPS) SINTER LAND INC., Chiyo, Japan). The heating rate was set to 100 °C/min for 0–600 °C, 50 °C/min for 600–900 °C, and 25 °C/min for 900–1100 °C. After sintering was completed, the samples were held at a temperature of 1100 °C with a pressure of 30 MPa for 10 min to inhibit the coarsening of the deleterious microstructures [24,25]. After cooling to room temperature, the cylindrical samples, with a diameter of 20 mm and a thickness of about 10 mm, were removed. The tensile and scanning specimens were then passed through a DK 7735 CNC wire-cutting machine (Taizhou Weihai CNC Machine Tool Co., Ltd., Taizhou, China). The samples were analyzed using a DX-2500 X-ray diffractometer (XRD, Dandong Haoyuan Instrument Co. Ltd., Dandong, China) with the following test conditions: Cu target Kα-rays, tube voltage: 40 kV, tube current: 40 mA, scanning angle range: 20–90°, step angle: 0.06°/s, and sampling time: 0.5 s. Surface micromorphology was determined by Quanta 450 FEG field emission scanning electron microscopy (FEI Company, Hillsboro, OR, USA). Room temperature tensile tests were performed by using an ETM-105 D universal testing machine (Shenzhen Wance Test Equipment Co., Ltd., Shenzhen, China) with the following test conditions: tensile specimens had an original scale distance of 5 mm, a cross-sectional area of 1.5 mm × 1 mm, and a tensile rate of 0.2 mm/min. Five samples of each alloy were tested to avoid errors. The hardness of the samples was measured by an MHVD 50 AP Vickers hardness tester (Shanghai Jujing Precision Instrument Co., Ltd., Shanghai Measurement Co., Ltd., Shanghai, China); the fixed load was 500 g, the retention time was 15 s, and the average of five points was measured for each sample. The density of the alloys was determined as the ratio of the density to the theoretical density and was measured based on the Archimedes method.

## 3. Results

### 3.1. CoCrFeNiNbx High-Entropy Alloy Powder

**Figure 1:**
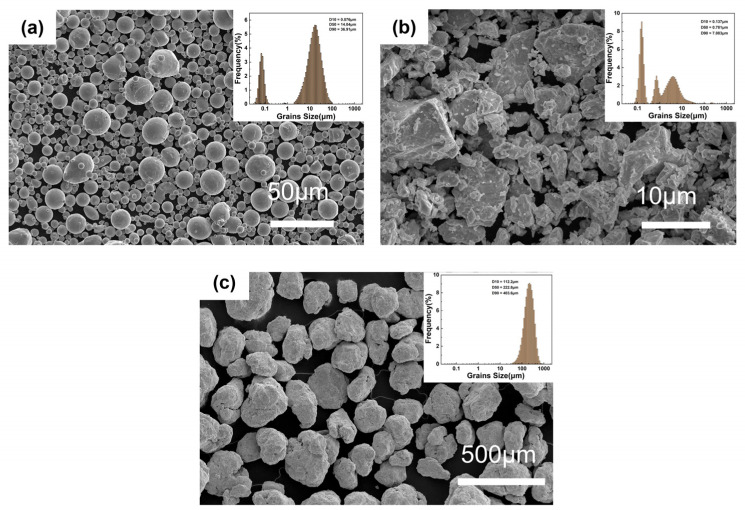
Various powders and their particle size distribution: (**a**) SEM of CoCrFeNi HEA powder, (**b**) SEM of Nb powder, and (**c**) SEM of CoCrFeNi-Nbx HEA powder.

Figure 1 demonstrates the SEM images of the CoCrFeNi, Nb, and ball-milled powders as well as particle size distribution images of the powders. Figure 1a shows the images of CoCrFeNi powders prepared by gas atomization, where smaller molten droplets experienced a higher cooling rate during gas atomization, and, therefore, had a shorter solidification time and were more likely to adhere to the surface of the larger molten droplets, leading to the observed satellite structure of some of the powders [25]. In addition, it can be noticed that the particle size of the powder after ball milling was much larger than that of the powder before ball milling, which is due to the phenomenon of powder agglomeration caused by the shorter milling time [26].

**Figure 2:**
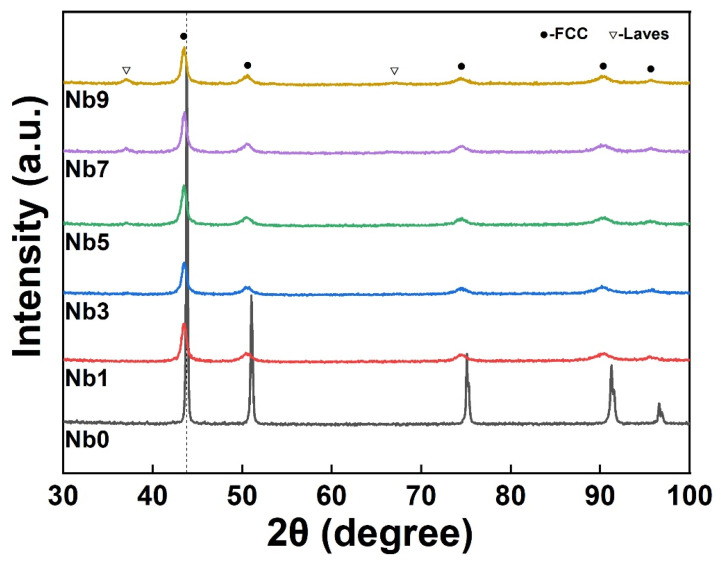
XRD patterns of the powders.

Figure 2 demonstrates the XRD image of the HEA powder after ball milling, and it can be clearly seen that the diffraction peaks of the powder after ball milling were shifted to a low angle, which was attributed to the fact that the diameter of the doped Nb atoms was larger than that of the four elements, Co, Cr, Fe, and Ni, which made the cellular parameter of the mixed powders larger and shifted the XRD diffraction peaks to a low angle [22]. Meanwhile, with the increase in Nb content, the diffraction peaks of the Laves phase appeared near 37° and 68°.

### 3.2. XRD Analysis

**Figure 3:**
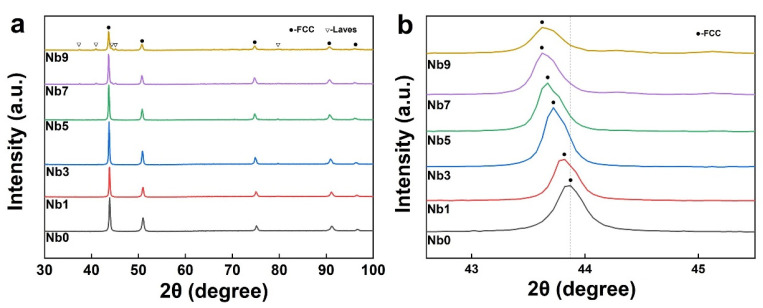
XRD patterns of the samples: (**a**) XRD patterns of CoCrFeNi-Nbx (x = 0, 1, 3, 5, 7, 9, wt%) HEAs and (**b**) XRD patterns of amplified peaks near 44°.

Figure 3 demonstrates the XRD patterns of the samples obtained by sintering CoCrFeNi-Nbx HEAs with different niobium contents as well as their magnification near 44°. As shown in Figure 3a, the niobium-free CoCrFeNi HEA had an FCC single-phase structure, and the addition of elemental Nb to CoCrFeNi HEA resulted in the formation of the Laves phase, which is a Co2Nb-type Laves phase with a hexagonal close-packed structure (HCP) [19]. With the increase in Nb content, it can be observed that the diffraction peaks of the Laves phase were gradually enhanced, and the intensity of the FCC diffraction peaks gradually decreased; moreover, the intensity of the peaks corresponding to the FCC phase was much higher than that of the Laves phase, indicating that the alloy was still dominated by the FCC phase. In addition, it can be found that with the increase in Nb content, the FCC peak near 44° was also gradually shifted to a lower angle, as in the case of ball-milled powder, shown in Figure 3b. The reason for this is the same as that of the leftward shift of the wave peaks after ball milling, i.e., due to the fact that the Nb atomic radius is larger than that of the four elements of CoCrFeNi, a lattice distortion effect is produced that leads to an increase in the cell parameters.

### 3.3. Microstructure

**Figure 4:**
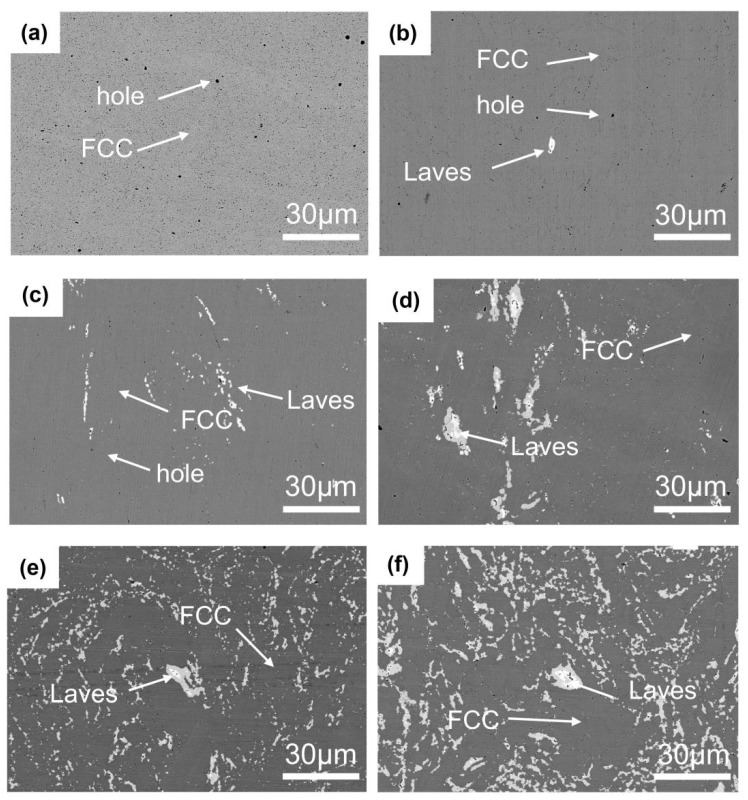
SEM image of CoCrFeNi HEA with different Nb contents. (**a**) CoCrFeNi HEA; (**b**) Nb1; (**c**) Nb3; (**d**) Nb5; (**e**) Nb7; and (**f**) Nb9.

**Figure 5:**
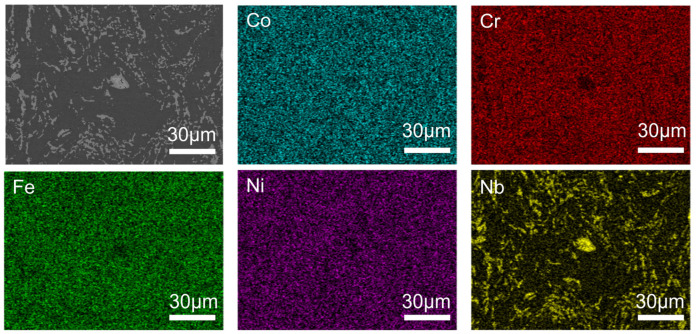
EDS scan results of CoCrFeNi-Nb9 HEA samples.

The SEM images of CoCrFeNi-Nbx HEAs with different niobium doping amounts after sintering are demonstrated in Figure 4a–d for Nb0, Nb1, Nb3, Nb5, Nb7, and Nb9, respectively. It can be seen that the percentage of the white Laves phase gradually increased with the increase in Nb content. In Figure 5a, i.e., when the niobium doping amount was 0%, a simple FCC solid solution structure is observed because the atomic sizes of the four elements, Co, Cr, Fe, and Ni, do not differ much and there is no obvious positive or negative mixing entropy between the atomic pairs. It is also not difficult to see that some very small voids can be observed on the surface of the sample, which is due to the inhomogeneity of the current during the SPS sintering process, which make the local current density at the sintering neck larger, the temperature too high, and the powder volatilize, resulting in the formation of micropores at higher sintering temperatures [25].

Table 1 demonstrates the results of the elemental content of different phases of alloys with different niobium contents. Combining the analysis of Figure 5 and Table 1, it can be concluded that the FCC phase was enriched with four elements, Fe, Co, Cr, and Ni, and that their mass proportions are close to each other, accounting for about 24% of the overall, with a lower content of Nb which increased with the percentage of Nb doped. In the Laves phase, the three elements of Cr, Fe, and Ni were depleted, with their contents decreasing from about 23% to 11–16%, and the elemental Nb was enriched by about 35%, while the Co element was not greatly affected.

Figure 5 demonstrates the result map obtained by the SEM scanning of the Nb9 sample, and it can be clearly seen that the content of the Nb element in the Laves phase is much higher than that in the FCC phase, which indicates that Nb is enriched in the Laves phase, the three elements of Cr, Fe, and Ni are impoverished, and the Co element is not much affected, which is in agreement with the study obtained in the previous section.

### 3.4. Mechanical Properties of Alloys

**Figure 6:**
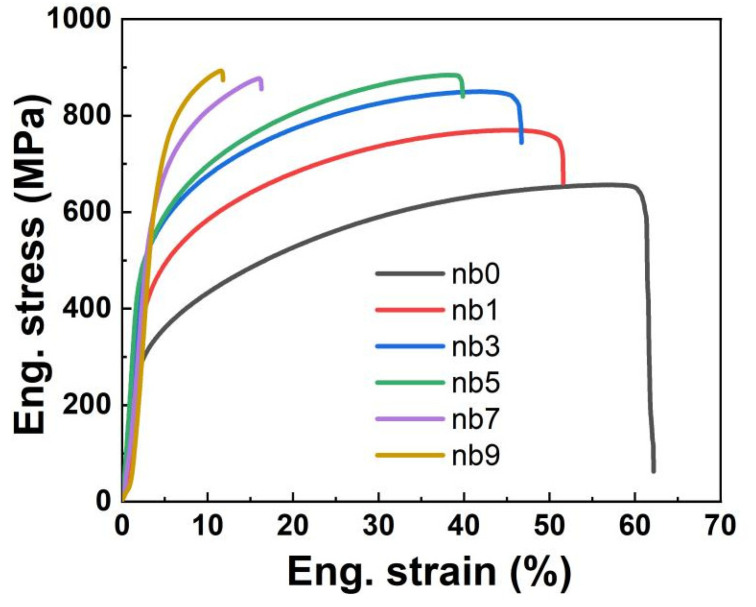
Room temperature engineering stress–strain curves for CoCrFeNi-Nbx HEAs at different Nb contents.

Figure 6 demonstrates the engineering stress–strain curves obtained by stretching CoCrFeNi-Nbx HEAs at room temperature, and it is easy to see that the tensile and yield strengths of the samples increase significantly with the increase in the Nb content, and the elongation decreases to some extent. The tensile properties of the HEAs are demonstrated in the following Table 2, showing that the yield and tensile strengths of the Nb0 were the lowest, which were 269.4 MPa and 656.3 MPa, respectively, and it had the highest elongation up to 62.2%. With the increase in Nb content, the yield strength and tensile strength of the HEA gradually increase and the elongation gradually decreases; at Nb9, the yield strength and tensile strength reach a maximum of 775.4 MPa and 893.2 MPa, respectively, while the elongation also decreases to the minimum of 11.8%. At the same time, it is easy to find that when the Nb content exceeds 5%, the tensile strength increases slightly while the elongation decreases considerably. The excellent tensile properties of the Nb5 alloy are attributed to a combination of the solid solution strengthening of the Nb atoms and second-phase hardening of the well-ordered Nb-rich Laves phase in the FCC matrix. The solid-solution Nb atoms greatly enhance the lattice distortion in the FCC matrix due to their large atomic size, and the formation of the ordered Nb-rich Laves phase significantly enhances the strength of the HEAs due to the precipitation strengthening effect, whereas the main-phase FCC matrix of Nb5 alloys provides good plasticity; the combination of the two results in the excellent overall tensile properties of Nb5 alloys [22]. However, as the Nb content continues to increase, many Laves phases impede the dislocation motion, resulting in a significant decrease in the plasticity of the alloy.

Table 3 shows the mechanical properties of CoCrFeNi HEAs doped with different Nb contents prepared by MA+SPS, compared with CoCrFeNi-Nbx HEAs prepared by arc melting, HEAs prepared by MA + SPS have more excellent properties. CoCrFeNi-Nbx HEAs prepared by arc melting by Rui Fan et al. have an ultimate tensile strength of 894 MPa, a plasticity of only 7.5% of that of CoCrFeNi HEAs, and a maximum yield strength of only 692 Mpa [27]. The plasticity of the sample prepared by W.H. Liu et al. was only 1.3%, although it possessed a larger ultimate tensile strength, and the plasticity of the sample whose tensile strength was 879 Mpa was only 3.5% [22]. On the other hand, the plasticity of the HEAs prepared in this paper still reached 11.8% at a tensile strength of 893.2 Mpa, and the maximum tensile strength and maximum yield strength were 893.2 Mpa and 775.4 Mpa, respectively, which are better than the samples prepared by arc melting.

**Figure 7:**
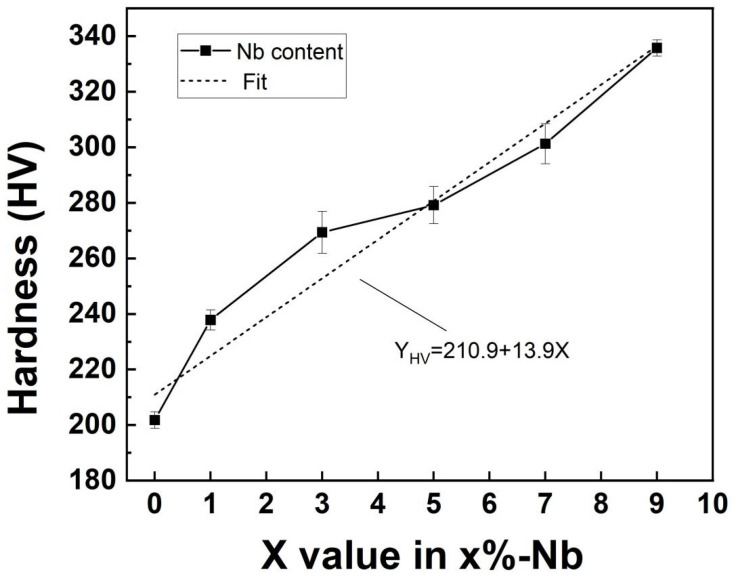
Hardness variation of CoCrFeNi-Nbx HEAs with different Nb contents.

Figure 7 demonstrates the variation of HEA hardness of CoCrFeNi-Nbx (x = 0, 1, 3, 5, 7, and 9 wt%) HEAs which increased from 201 HV for Nb0 to 336 HV for Nb9; the hardness of the alloys was almost proportional to the content of Nb. The reason for this is that the increase in Nb content leads to an increase in the volume fraction of the Laves phase, and the increase in the hard Laves phase leads to an increase in the HEA hardness.

**Figure 8:**
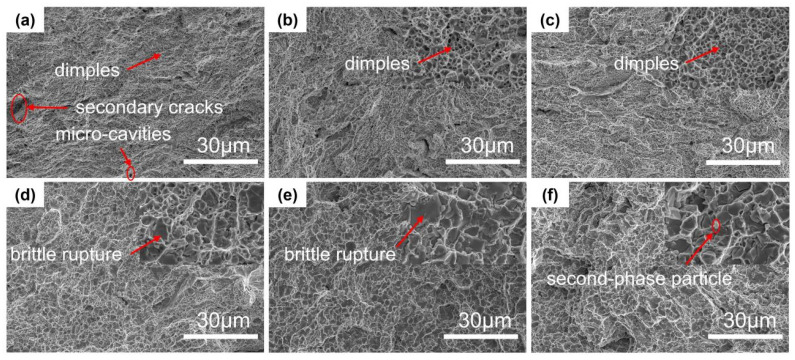
SEM images of the tensile fracture of CoCrFeNi-Nbx HEAs with different niobium contents. (**a**) CoCrFeNi HEA; (**b**) Nb1; (**c**) Nb3; (**d**) Nb5; (**e**) Nb7; and (**f**) Nb9.

Figure 8 shows the morphology of the tensile fracture of CoCrFeNi-Nbx (x = 0, 1, 3, 5, 7, and 9 wt%) HEAs; it can be found that when the Nb content is low, obvious dent characteristics can be observed on the sample, indicating that the sample has undergone considerable plastic deformation and has a ductile fracture. At a Nb content of 5, the ductile fracture range is slightly larger than the brittle fracture range, and gives the best overall performance, whereas at Nb contents of 7 and 9, the brittle fracture occupies most of the range, and, hence, the plasticity of the HEA is drastically reduced. This is consistent with the changes in the tensile properties of HEAs produced with increasing Nb content observed in Figure 6. In addition, more secondary cracks and micropores can be observed in Figure 8a,b, which is also consistent with its highest elongation.

**Figure 9:**
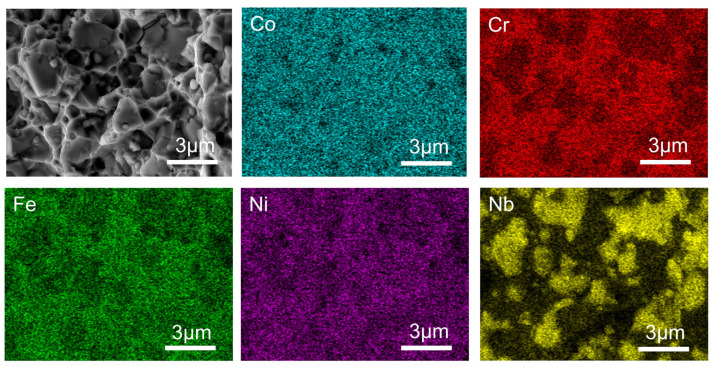
EDS scan results of Nb9 HEA tensile fracture.

Meanwhile, by the EDS scanning of the Nb9 alloy fracture, it was found that the brittle fracture region was enriched with Nb elements at a much higher content, while Fe, Cr, and Ni elements were depleted, as shown in Figure 9. This indicates that the Nb element leads to the generation of a hard Laves phase, resulting in the brittle fracture of the HEA, which is consistent with the results of the previous study.

## 4. Conclusions

In this paper, CoCrFeNi-Nbx (x = 0, 1, 3, 5, 7, and 9 wt%) HEAs were prepared by high-energy ball milling and discharge plasma sintering methods, and the effect of the Nb elements on the CoCrFeNi HEAs produced was systematically investigated. At the same time, the results were compared with those of the CoCrFeNi-Nbx HEAs prepared by the arc melting method as follows:

1. The XRD results show that with the addition of the Nb element, the lattice distortion of the alloy due to the large size of Nb atoms causes the microstructure of the CoCrFeNi HEAs to change from a single-phase FCC structure to a dual-phase structure of FCC and Laves.
2. With the increase in the Nb content, the volume fraction of the hard and brittle phase Laves phase increases, leading to an enhancement of the HEA’s tensile strength, yield strength, and hardness, and a decrease in its plasticity. Among them, the Nb5 HEA showed the most excellent overall performance, with a tensile strength, yield strength, and plasticity of 879.1 Mpa, 491.8 Mpa, and 39.8%, respectively. All the performances were enhanced compared with those of the HEAs obtained by the arc melting method.
3. The increase in the HEA hardness is nearly proportional to the increase in the volume fraction of the Laves phase, and the Laves phase is the direct cause of the increase in HEA hardness.
4. More toughness can be observed when the Nb element content is low, indicating that the HEA is still dominated by the toughness fracture. When the Nb content is higher, then basically no toughness can be observed and the HEA is dominated by brittle fractures.

## Figures and Tables

**Table 1:** Mixing enthalpy of different atom pairs ${∆H}_{AB}^{mix}$ (kJ/mol) calculated by Miedema’s approach [23].

Element (Atomic Radius, Melting Point)	Co	Cr	Fe	Ni	Nb
Co (0.1251 nm, 1495 °C)	-	−4	−1	0	−25
Cr (0.1249 nm, 1857 °C)	-	-	−1	−7	−7
Fe (0.1241 nm, 1535 °C)	-	-	-	−2	−16
Ni (0.1246 nm, 1453 °C)	-	-	-	-	−30
Nb (0.1429 nm, 1950 °C)	-	-	-	-	-

**Table 2:** EDS analysis results of CoCrFeNi-Nbx HEAs with different Nb contents in sintered conditions.

Alloys	Phase	Chemical Composition/at.%				
		Co	Cr	Fe	Ni	Nb
Nb0	FCC	26.05	23.74	24.87	24.34	0
Nb1	FCC	25.68	23.16	24.7	25.21	1.25
	Laves	24.16	12.04	14.35	15.83	33.62
Nb3	FCC	25.25	22.49	23.99	24.86	3.42
	Laves	23.8	11.21	14.49	15.97	34.53
Nb5	FCC	24.73	21.91	23.34	24.04	5.98
	Laves	22.82	11.8	14.47	16.06	34.86
Nb7	FCC	24.05	21.53	22.92	23.79	7.71
	Laves	23.27	11.52	14.66	16.12	34.43
Nb9	FCC	23.53	21.04	22.39	23.25	9.79
	Laves	22.28	11.73	14.5	16.09	35.4

**Table 3:** Mechanical properties of CoCrFeNi HEAs doped with different Nb contents prepared by MA+SPS.

Alloys	Yield Strength (Mpa)	Ultimate Tensile Strength (Mpa)	Elongation (%)	Density (%)
Nb0	269.4	656.3	62.2	99.2 ± 0.4
Nb1	393.5	769.8	51.6	98.5 ± 0.8
Nb3	487.9	849.9	46.7	99.0 ± 0.6
Nb5	491.8	879.1	39.8	98.8 ± 0.6
Nb7	624.8	875.8	16.3	99.0 ± 0.3
Nb9	775.4	893.2	11.8	99.3 ± 0.5

## Data Availability

Data are contained within the article.
